# The mRNA and miRNA profiles of goat bronchial epithelial cells stimulated by *Pasteurella multocida* strains of serotype A and D

**DOI:** 10.7717/peerj.13047

**Published:** 2022-03-18

**Authors:** Qi An, Si Chen, Luyin Zhang, Zhenxing Zhang, Yiwen Cheng, Haotian Wu, Ang Liu, Zhen Chen, Bin Li, Jie Chen, Yiying Zheng, Churiga Man, Fengyang Wang, Qiaoling Chen, Li Du

**Affiliations:** Hainan Key Lab of Tropical Animal Reproduction, Breeding and Epidemic Disease Research, Animal Genetic Engineering Key Lab of Haikou, College of Animal Science and Technology, Hainan University, Haikou, Hainan, China

**Keywords:** mRNA-Seq, miRNA-Seq, *Pasteurella multocida*, Goat bronchial epithelial cells, Immune response

## Abstract

*Pasteurella multocida* (*P. multocida*) is a zoonotic bacterium that predominantly colonizes the respiratory tract and lungs of a variety of farmed and wild animals, and causes severe respiratory disease. To investigate the characteristics of the host immune response induced by *P. multocida* strains of serotype A and D, high-throughput mRNA-Seq and miRNA-Seq were performed to analyze the changes in goat bronchial epithelial cells stimulated by these two serotypes of *P. multocida* for 4 h. Quantitative RT-PCR was used to validate the randomly selected genes and miRNAs. The results revealed 204 and 117 differentially expressed mRNAs (|log_2_(Fold-change)| ≥ 1, *p-value* < 0.05) in the *P. multocida* serotype A and D stimulated groups, respectively. Meanwhile, the number of differentially expressed miRNAs (|log_2_(Fold-change)| > 0.1, *p-value* < 0.05) were 269 and 290, respectively. GO and KEGG enrichment analyses revealed 13 GO terms (*p-value* < 0.05) and four KEGG pathways (*p-value* < 0.05) associated with immunity. In the serotype A-stimulated group, the immune-related pathways were the GABAergic synapse and Toll-like receptor signaling pathways, while in the serotype D-stimulated group, the immune-related pathways were the phagosome and B cell receptor signaling pathways. Based on the predicted results of TargetScan and miRanda, the differentially expressed mRNA–miRNA network of immune-related GO terms and KEGG pathways was constructed. According to the cell morphological changes and the significant immune-related KEGG pathways, it was speculated that the *P. multocida* serotype D strain-stimulated goat bronchial epithelial cells may induce a cellular immune response earlier than serotype A-stimulated cells. Our study provides valuable insight into the host immune response mechanism induced by *P. multocida* strains of serotype A and D.

## Introduction

*Pasteurella multocida* is a common pathogen that infects humans and a variety of farmed and wild animals. This organism threatens human health as well as global animal husbandry (Mogilner & Katz, 2019). For ruminants, including goats, sheep, and cattle, serotypes of *P. multocida* can cause various clinical manifestations (Harper et al., 2011), and the pathogenic mechanisms mediated by *P. multocida* in different animals are complex. Pathogenic differences relate to variations in virulence factors and structural modifications among serotypes (Peng et al., 2019). Thus, it is essential to elucidate the specific pathogenic mechanism of *P. multocida* according to the serotype of the infecting strain.

According to capsule type, *P. multocida* can be divided into five serotypes: A, B, D, E, and F (Heddleston, Gallagher & Rebers, 1972). During *P. multocida* infection, virulence factors, including lipopolysaccharide, capsule, toxin, adhesion factor, iron metabolism-related protein, hyaluronidase, outer membrane protein, and sialic acid metabolism-related protein, play variable roles in pathogenesis (Wilkie et al., 2012). Boyce and Adler demonstrated that *P. multocida* is rapidly eliminated in the host on loss of its capsule (Boyce & Adler, 2000). Harper and colleagues observed that *pcgC* mutant of *P. multocida* VP161 strain resulted in fewer bacteria in infected chickens (Harper et al., 2007). Sellyei and coworkers proposed that *ptfA* gene, which encodes the fimbriae of *P. multocida*, plays an indispensable role in the adhesion of bacteria to host epithelial cells (Sellyei, Bányai & Magyar, 2010; Tang et al., 2009). Different virulence factors cause distinct host responses dependent on the serotypes of *P. multocida*.

In China, *P. multocida* serotype A, B, and D are the most prevalent. Epidemiological studies suggest that *P. multocida* serotypes A and D are commonly associated with fowl cholera, conjunctivitis, and respiratory disorders such as rhinitis and pneumonia, while serotype B is more frequently associated with hemorrhagic septicemia (Peng et al., 2019). Therefore, we selected *P. multocida* serotype A and D strains isolated from sheep and goat to explore the differences in host immune responses induced by these strains.

The target cells of *P. multocida* infection include spleen cells, liver cells, lung cells, macrophages, and epithelial cells (Kubatzky, 2012). Epithelial cells form an indispensable physical barrier (Wilson & Ho, 2013). They produce antimicrobial peptides and activate innate immune receptors, which effectively resist bacterial invasion. During *P. multocida* invasion of the host bronchus, adhesion to epithelial cells is vital to successful colonization and subsequent infection (Marques & Boneca, 2011). Sudaryatma and colleagues reported that *P. multocida* can adhere to human alveolar basal epithelial type II (A549) cells. When epithelial cells are infected by bovine respiratory syncytial virus, the adhesion ability of *P. multocida* can be increased 2–8-fold (Sudaryatma et al., 2018). This suggests that respiratory epithelial cell is an important physical barrier against *P. multocida* infection.

Zamri-Saad and coworkers established a model of pasteurellosis pneumonia in goats using *P. multocida* isolated from the lungs of goats, sheep, and rabbits. Goats are considered a good model in the study of pasteurellosis pneumonia, especially regarding the vaccination or pathogenesis of *P. multocida* (Zamri-Saad et al., 1996). Considering that the mechanisms involved in the host–bacteria interaction between goat bronchial epithelial cells and invading *P. multocida* remain unclear, we used goat bronchial epithelial cells to construct an infection model of *P. multocida*.

Transcriptome studies allow for gene transcription and transcriptional regulation to be studied *in vivo* or *in vitro* at the RNA level (Costa et al., 2010; Wang, Gerstein & Snyder, 2009). Compared with traditional Sanger sequencing technology, high-throughput sequencing technology is more sensitive, rapid, reproducible, and enables greater coverage (Cloonan et al., 2008; Morin et al., 2008; Nagalakshmi et al., 2008). RNA-Seq has been widely used to explore the mechanisms involved in host–bacteria interactions. Through comparative analysis of transcriptomes, Aprianto and colleagues found that pneumococcal adhesion inhibited the expression of *IL-8* gene in human lung epithelial cells (Aprianto et al., 2016). Wu and coworkers established a *P. multocida*-induced pneumonia mouse model and transcriptome sequencing showed that the expression of the *IL-17* gene was significantly upregulated in the lungs of *P. multocida*-infected mice (Wu et al., 2017). In our previous study, transcriptome sequencing and small RNA sequencing on goats challenged with *P. multocida* identified 2,673 significantly differentially expressed mRNAs and 56 miRNAs, some of them are involved in inflammatory response, immune effector process, and cell activation in response to endogenous stimuli (Chen et al., 2021).

In this study, we performed sequence analysis to investigate the effects of *P. multocida* infection with serotype A and D strains on mRNA and miRNA expression of goat bronchial epithelial cells *in vitro*.

## Materials & Methods

### Bacterial strains and culture conditions

The *P. multocida* strains used in this study were previously isolated and stored by Hainan Key Lab of Tropical Animal Reproduction, Breeding and Epidemic Disease Research (Cao et al., 2018). Briefly, serotype A *P. multocida* HN02 strain (GenBank Accession Number: CP037865.1) and serotype D *P. multocida* HN01 (GenBank Accession Number: CP037861.1) were isolated from the lung tissue of a sheep and a Hainan black goat, respectively, both of which had died of pneumonia. The strains were confirmed by amplifying the 16S rRNA using the primers: hyaD-hyaC (serotype A specific primers) and dcbF (serotype D specific primer). The PCR cycling conditions were as follows: an initial denaturation of 5 min at 95 °C, then 30 cycles of 50 s at 95 °C, 1.5 min at 60 °C, and 1.5 min at 72 °C, followed by 5 min at 72 °C. The products were detected by agarose gel electrophoresis. The bacterial strains were cultured in Tryptic Soy Broth (TSB) (Hopebio, China) supplemented with 5% (v/v) bovine serum (Sijiqing, China) at 37 °C overnight and were stored at −20 °C in glycerol at a final concentration of 12.5%. The resuscitated bacterial strains were identified according to the above steps.

### Cell culture

Goat bronchial epithelial cells were obtained from a healthy 7-month-old female goat (iCell Bioscience Inc., Shanghai, China). Cells were cultured in 25-cm^2^ cell culture flasks in 5% CO_2_ at 37 °C. The medium was epithelial cell culture medium containing 2% fetal bovine serum (FBS) (PriMed-iCell-001, iCell Bioscience Inc.). Passage was carried out when the confluence of cells was >85%. In brief, the cultured cells were washed twice with sterile phosphate-buffered saline (PBS), then digested with trypsin (HyClone, Logan, Utah, USA). Digestion was stopped by adding RPMI 1640 medium (Sangon Biotech, Shanghai, China) containing 10% FBS. Cells were then centrifuged, resuspended in new culture flasks, and cultured.

### *P. multocida* stimulation experiments

The stored *P. multocida* serotype A and D strains were resuscitated on soybean-casein digest agar medium (TSA) (Hopebio, China) containing 10% (v/v) bovine serum at 37 °C overnight, and then monoclonal colonies were inoculated into 20 mL of TSB. Based on the pre-determined growth curves and standard linear equations of the two strains (Figs. S1 and S2), cultures with an optical density (OD) of 0.6 at 600 nm (approximately 2 × 10^9^ CFU/mL) were used to prepare infection suspensions with a multiplicity of infection (MOI) of 100. Goat bronchial epithelial cells were then incubated with one mL of infection suspension for 4 h after three passages, whereas cells in the control group were added to one mL of epithelial cell culture medium containing 10% FBS (Gibco, Invitrogen, USA). Extracellular bacteria were removed by washing three times with sterile PBS. After rinsing three times with primary epithelial cell culture medium containing 10% FBS and 200 µg/mL gentamycin, cells were washed again with PBS and harvested with Trizol (Invitrogen, Carlsbad, CA, USA). The lysates were frozen in liquid nitrogen. The experiments were repeated three times.

### mRNA library construction and sequencing

Nine cell samples from three different treatments, designated Ctrl_1/2/3, A_T4 h_1/2/3, and D_T4 h_1/2/3, were sent to Lc-Bio Technologies Co., Ltd. (Hangzhou, China) for mRNA-Seq analysis. In brief, total RNA from each sample was extracted using Trizol reagent following the manufacturer’s procedure. RNA quantity and purity were confirmed using an Agilent 2100 Bioanalyzer (Agilent technologies Santa Clara, CA, USA) with RNA 1000 Nano LabChip kit (Agilent, CA, USA). Next, mRNAs were enriched using oligo(dT)-attached magnetic beads and fragmented into small pieces using divalent cations under an elevated temperature. A reverse transcription reaction was performed to obtain double-stranded cDNA. Following cDNA purification with AMPure XP beads, T4 and Klenow DNA polymerases were used to generate blunt ends. Modifications to the 3′ ends included adenylation and adapt ligation. AMPure XP beads were used to select fragments, followed by PCR to obtain the cDNA library. Paired-end sequencing was performed on an Illumina Novaseq™ 6000 (Illumina, San Diego, CA, USA) following the manufacturer’s protocol. The raw data were deposited into the NCBI Sequence Read Archive (SRA) under accession number PRJNA655134.

### miRNA library construction and sequencing

MiRNA library construction and sequencing were carried out as previously described in Pang et al. (2019) study. The raw data were deposited into the NCBI SRA under accession number PRJNA655261.

### Bioinformatic analysis of mRNA sequencing data

To obtain valid data (clean data), the raw data were processed by cutadapt software (Version 1.9) to filter out the low-quality reads, as well as reads with adapters, reads containing more than 5% unknown nucleotides (N). The valid data were then mapped to the reference genome (GCF_001704415.1) using Hisat (Version 2.0) and information was recorded, including read statistics, regional distribution, and principal component analysis (PCA). StringTie (Version 1.3.0) was used to analyze the expression levels of genes by calculating fragments per kilobase of the exon model per million mapped reads (FPKM). The selected standards for differentially expressed genes between different treatment groups were —log_2_ (Fold-change)— ≥ 1 and statistical significance (*p-value*<0.05). The Kyoto Encyclopedia of Genes and Genomes (KEGG) database (http://www.kegg.jp/kegg) and Gene Ontology (GO) database (http://geneontology.org) were used for pathway annotation of the differentially expressed genes.

### Bioinformatic analysis of miRNA sequencing data

The analysis of miRNA was carried out according to the method of Li et al.’s (2021a). And a previously reported method (Li et al., 2016; Cer et al., 2014) was used to normalize the expression levels of miRNA. MiRNAs with —log_2_ (Fold-change)— > 0.1 and *p-value* < 0.05 were regarded as differentially expressed.

### Targeted prediction of differentially expressed miRNAs

TargetScan (Version 3.3a) and miRanda (Version 3.3a) software were used to predict the target genes of miRNAs with significant differences. The predicted target genes were screened as follows. In TargetScan, the target genes with a context score percentile less than 50 were removed, while in the miRanda algorithm, target genes with a max free energy greater than −10 were removed. The common target genes predicted by both two softwares were screened for the final target genes of differentially expressed miRNAs.

### qRT-PCR validation

To validate the differentially expressed genes and miRNAs from multiple samples, qRT-PCR was performed using the previously extracted total RNA. Briefly, following removal of the genomic DNA from the total RNA, first-strand cDNA was synthesized using the RevertAid First Strand cDNA Synthesis Kit (Thermo Fisher Scientific, MA, USA) according to the manufacturer’s protocol. Genes and miRNAs were randomly selected for qRT-PCR validation. Primers were designed using the Primer Premier software (Version 5.0, PRIMER Biosoft International, Palo Alto, CA, USA) and the sequences are shown in Table 1. The qRT-PCR was performed using a SYBR^®^ Select Master Mix (ABI, CA, USA), and the following was the thermal cycle profile: pre-degeneration at 95 °C for 10 min, then 40 cycles of 95 °C for 15 s, 60 °C for 1 min, followed by 95 °C for 15 s (tested once every 0.3 °C temperature rise). Expression levels of mRNAs and miRNAs were normalized to GAPDH and U6 expression, respectively. Reactions were performed in triplicate and one reaction for each gene was randomly selected for agarose gel electrophoresis. StepOne software (Version 2.3) was used along with the Δ ΔCT method for data analysis.

**Table 1:** Primers for gene and miRNA expression analysis by qRT-PCR.

Name	Primer Sequence (5′–3′)	Tm (°C)	Size (bp)
IRF7	F: GCAAAGTCTACTGGGAGGTGG	61.1	101
	R: GAAGTCAAAGATGGGCGTGTC	62	
IL1A	F: AATAATCTGGAGGAGGCA	53	138
	R: CTTTAGCAAGACGGGTTC	53.4	
TNFSF18	F: TGGCTCCTCTACTCAACA	52.3	110
	R: GGTCCAAACTTCGCTACA	54.6	
SPP1	F: ATGATGGCCGAGGTGATA	57.3	180
	R: TGGTTTCTTCGCTGTGGT	57.7	
DUSP4	F: AGCACAGCGGAGTCTTTGGA	63.4	190
	R: CGAAGTGGTTTGGGCAGTCA	64.5	
IL12RB2	F: TGTTCACTGGCACTTACTT	51.5	153
	R: GCCTTGTTTGGGCTTCA	58.2	
IL6R	F: GGCAACATCTCAGTCAGCG	60.6	197
	R: CCACTCCAGGCATCACG	59.7	
CARD14	F: ACTTGAGCCAGGAGGAGTATGAC	61	136
	R: AGGAGGGCGTGGGTGTTCT	64.3	
GAPDH	F: CTCTCTGCTCCTGCCCGTTC	64.8	241
	R: TGTGCCGTGGAACTTGCCAT	67.1	
Chi-let-7i-5p	F: ACACTCCAGCTGGGTGAGGTAGTAGTTTGT	69.1	72
	R: CTCAACTGGTGTCGTGGAGTCGGCAATTCAGTTGAGAACAGCAC	86.8	
Chi-miR-15b-3p	F: ACACTCCAGCTGGGCGAATCATTATTTGC	73.8	71
	R: CTCAACTGGTGTCGTGGAGTCGGCAATTCAGTTGAGAGAGCAGC	87.4	
bta-miR-138	F: ACACTCCAGCTGGGAGCTGGTGTTGTGAATC	76.6	73
	R: CTCAACTGGTGTCGTGGAGTCGGCAATTCAGTTGAGCGGCCTGA	90.8	
bta-miR-2285i_R+1_1SS6CT	F: ACACTCCAGCTGGGAAAACTGGAACGAACTT	74.6	73
	R: CTCAACTGGTGTCGTGGAGTCGGCAATTCAGTTGAGGCCAAAAA	87.8	
U6	F:CTCGCTTCGGCAGCACA	59.3	94
	R: AACGCTTCACGAATTTGCGT	60.8	
URP	TGGTGTCGTGGAGTCG	49.4	

## Results

### Changes in cell morphology

**Figure 1:**
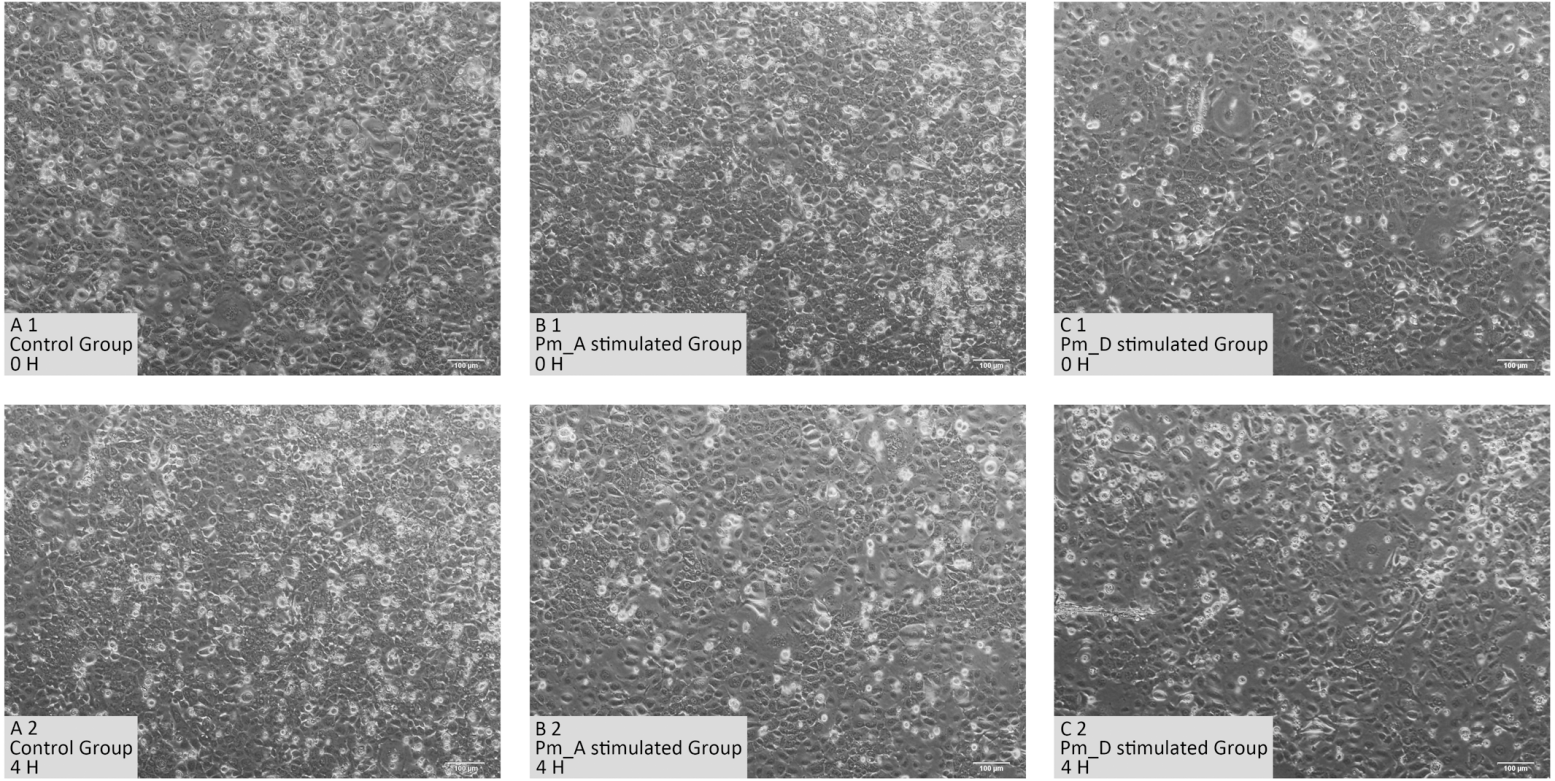
Cellular changes in goat bronchial epithelial cells stimulated with *P. multocida* strains of serotype A and D strains for 4 h. (A1)–(C1) Cell morphology of unstimulated group and stimulated groups at 0 h. (A2)–(C2) Cell morphology of unstimulated group and stimulated groups at 4 h.

As shown in Fig. 1, normal goat bronchial epithelial cells are cobblestone-like adherent cells. After 4 h stimulation with the *P. multocida* strain of serotype A, the intercellular space became larger and some cells became rounded and non-adherent. When stimulated with the *P. multocida* strain of serotype D, more cells were non-adherent and showed irregular morphology.

### Quality checking of RNA-seq data

**Figure 2:**
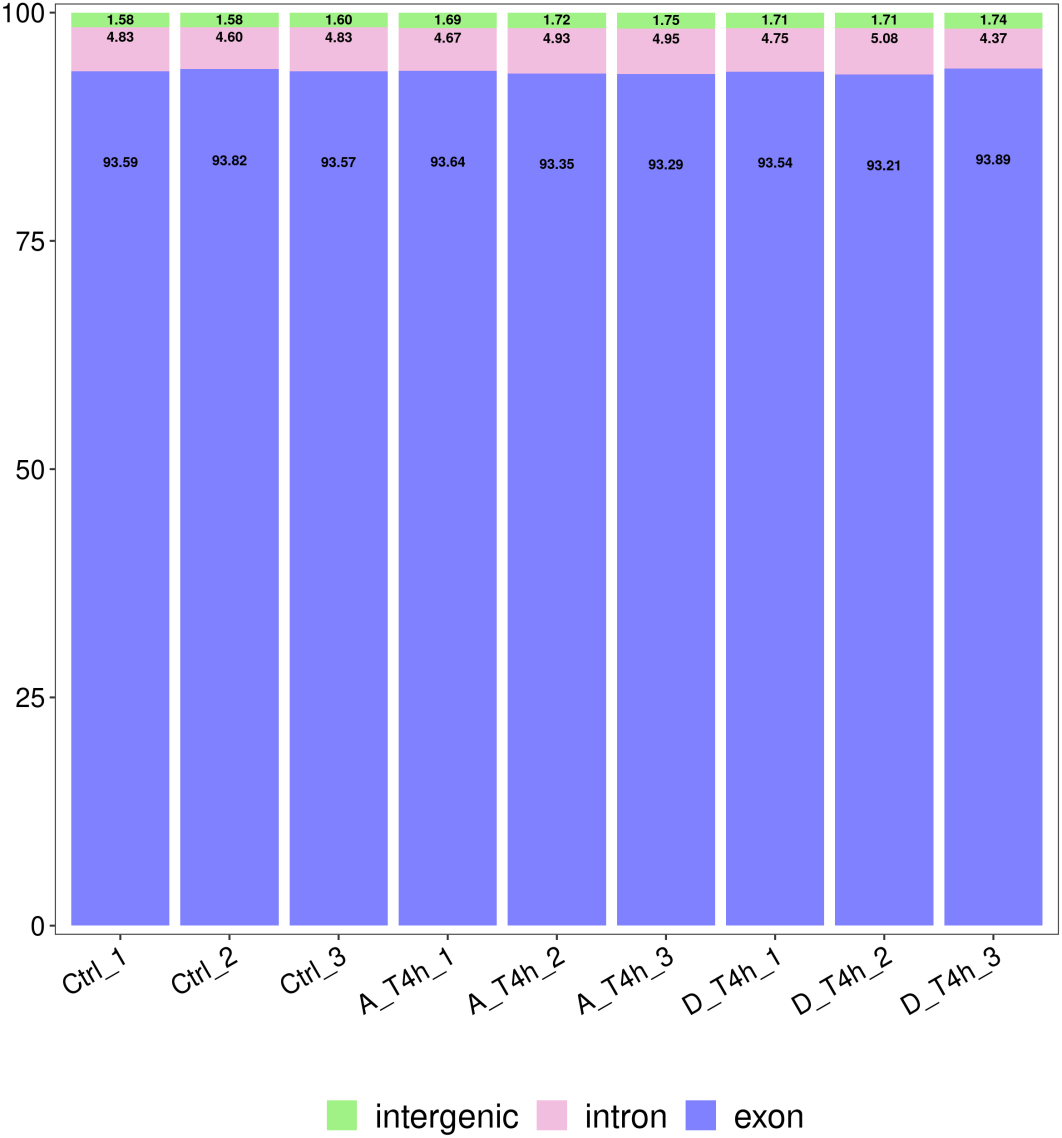
Statistical analysis of the genomic structure of valid reads. The vertical axis shows the proportions of the three gene structures.

**Figure 3:**
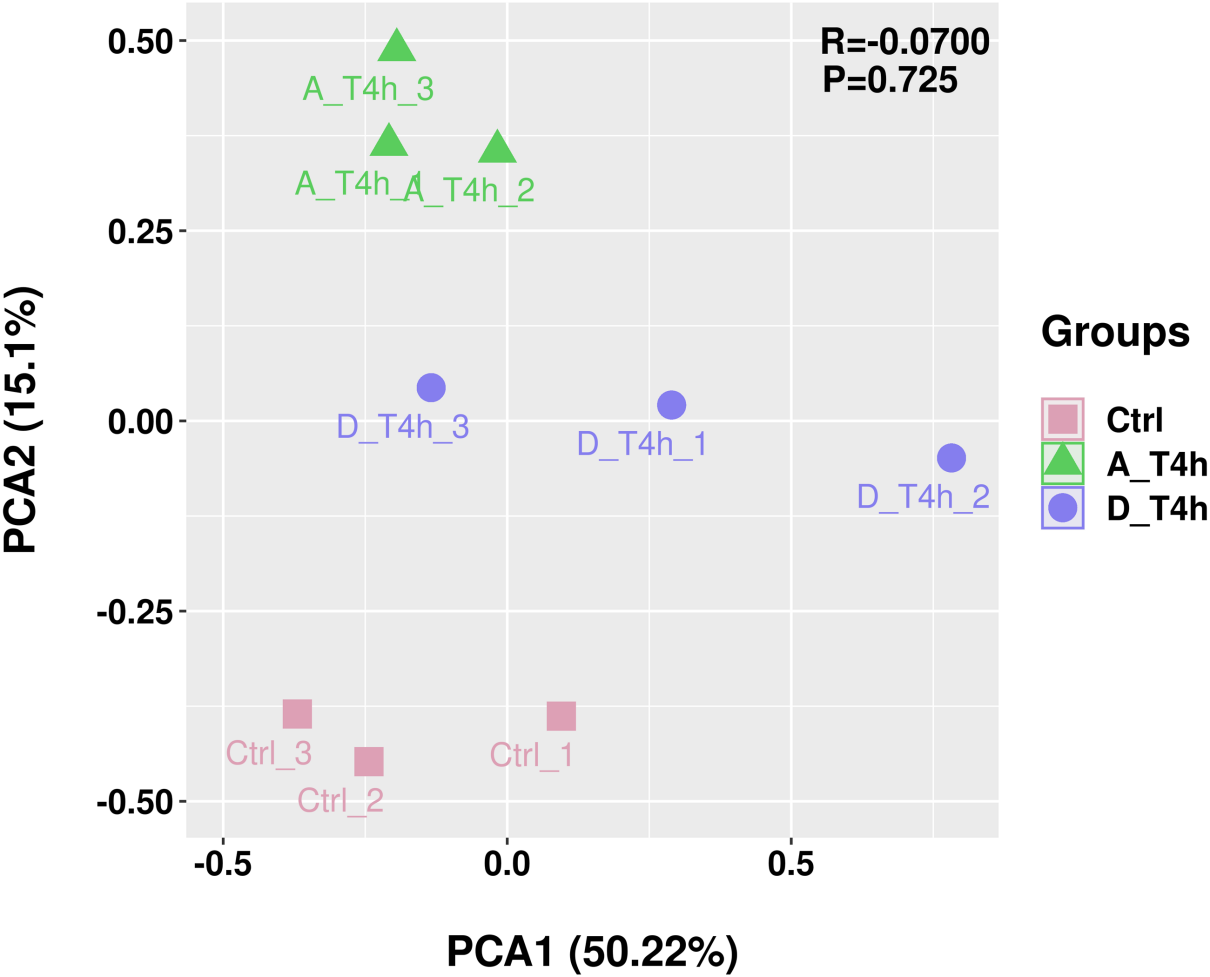
Principal component analysis (PCA) plot. Different colors and shapes represent different groups, each with three replicates.

The statistical power of the experimental design was checked using RNASeqPower (https://bioconductor.org/packages/release/bioc/html/RNASeqPower.html) and was 0.98. The amount of raw data generated by each sample is shown in Table 2. After filtering, 25,883,644-47,830,984 valid data were obtained from each sample and the valid ratios were all above 96.5% (Table 2). As shown in Table 3, after comparison with the reference genome, the number of mapped reads was above 97%, which indicates the samples were of high quality. In addition, statistical analysis of the genomic structure of valid reads showed that exons accounted for above 93% (Fig. 2). PCA was used to reduce the dimension of the sequencing data (Fig. 3). It showed that the 1st principal component (PC1) contained 50.22% of the variance, while the 2nd principal component (PC2) contained 15.1% of the variance. Our PCA results revealed that after *z*-score normalization processing, the samples from the same treatment clustered together, whereas the control group and the two experimental groups were separate. It confirmed the reproducibility of the samples.

**Table 2:** Statistical results for the RNA raw data.

Sample	Raw data		Valid data		Valid ratio (reads)	Q20%	Q30%	GC content%
	Read	Base	Read	Base				
Ctrl_1	40010384	6.00G	38763418	5.81G	96.88	99.98	98.18	48
Ctrl_2	46491232	6.97G	45219788	6.78G	97.27	99.98	98.02	48
Ctrl_3	49210810	7.38G	47830984	7.17G	97.20	99.98	98.17	48
A_T4 h_1	47608500	7.14G	46391112	6.96G	97.44	99.98	97.95	48
A_T4 h_2	44011732	6.60G	42667940	6.40G	96.95	99.98	98.07	47.50
A_T4 h_3	47924786	7.19G	46453594	6.97G	96.93	99.98	98.12	47
D_T4 h_1	36983278	5.55G	35864016	5.38G	96.97	99.98	98.10	48
D_T4 h_2	26730382	4.01G	25883644	3.88G	96.83	99.98	98.29	47
D_T4 h_3	45917970	6.89G	44608748	6.69G	97.15	99.98	98.02	48

**Table 3:** Statistics for the reference genome alignments.

Sample	Valid reads	Mapped reads	Unique mapped reads	Multi mapped reads	PE mapped reads	Reads map to sense strand	Reads map to antisense strand	Non- splice reads	Splice reads
Ctrl_1	38763418	37807871 (97.53%)	29868004 (77.05%)	7939867 (20.48%)	33206340 (85.66%)	17225038 (44.44%)	17241395 (44.48%)	18910088 (48.78%)	15556345 (40.13%)
Ctrl_2	45219788	44078884 (97.48%)	34709549 (76.76%)	9369335 (20.72%)	39391476 (87.11%)	20125676 (44.51%)	20143520 (44.55%)	22056988 (48.78%)	18212208 (40.27%)
Ctrl_3	47830984	46684392 (97.60%)	36836297 (77.01%)	9848095 (20.59%)	41250982 (86.24%)	21183939 (44.29%)	21203482 (44.33%)	23172307 (48.45%)	19215114 (40.17%)
A_T4 h_1	46391112	45138922 (97.30%)	35482143 (76.48%)	9656779 (20.82%)	40177014 (86.60%)	20497427 (44.18%)	20517519 (44.23%)	22870956 (49.30%)	18143990 (39.11%)
A_T4 h_2	42667940	41515873 (97.30%)	32665549 (76.56%)	8850324 (20.74%)	36387522 (85.28%)	18850948 (44.18%)	18868646 (44.22%)	21133437 (49.53%)	16586157 (38.87%)
A_T4 h_3	46453594	45235688 (97.38%)	35554191 (76.54%)	9681497 (20.84%)	39646378 (85.35%)	20559852 (44.26%)	20578799 (44.30%)	23319895 (50.20%)	17818756 (38.36%)
D_T4 h_1	35864016	34962146 (97.49%)	27584731 (76.91%)	7377415 (20.57%)	30704328 (85.61%)	15905496 (44.35%)	15922287 (44.40%)	17892395 (49.89%)	13935388 (38.86%)
D_T4 h_2	25883644	25253083 (97.56%)	20025119 (77.37%)	5227964 (20.20%)	21647082 (83.63%)	11457350 (44.26%)	11467649 (44.30%)	12921853 (49.92%)	10003146 (38.65%)
D_T4 h_3	44608748	43465480 (97.44%)	34140391 (76.53%)	9325089 (20.90%)	38273354 (85.80%)	19693887 (44.15%)	19712295 (44.19%)	22384493 (50.18%)	17021689 (38.16%)

### Differential expression analyses of mRNAs and miRNAs

**Figure 4:**
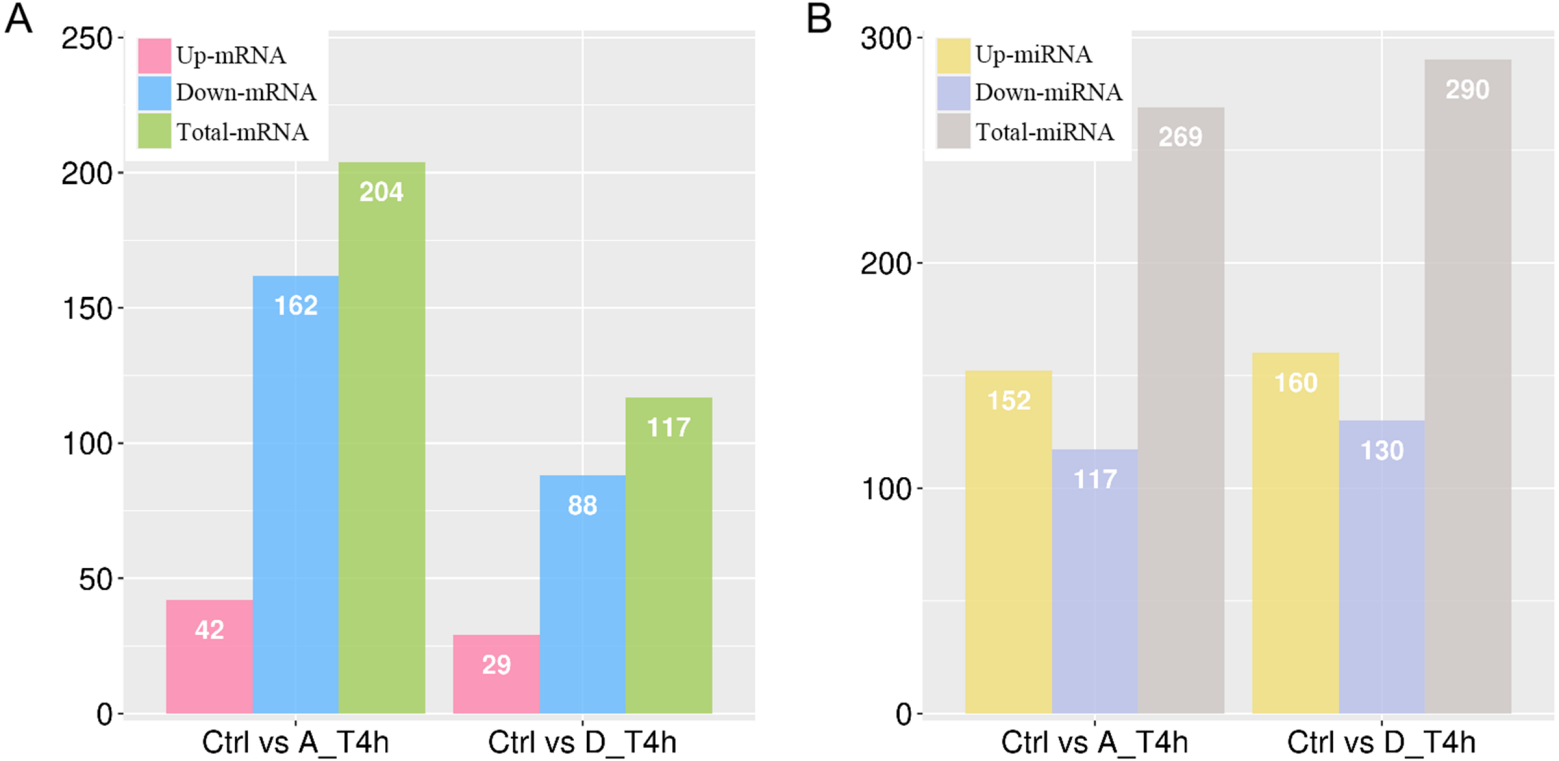
The numbers of differentially expressed mRNAs (A) and miRNAs (B) in *P. multocida* strain serotype A and D stimulated goat bronchial epithelial cells. (A) and (B) The number of upregulated and downregulated differentially expressed mRNAs and miRNAs in the two groups.

Based on the criterion of —log_2_ (Fold-change)— ≥ 1 and a *p-value* < 0.05, 204 (42 upregulated and 162 downregulated) and 117 (29 upregulated and 88 downregulated) differentially expressed mRNAs were identified in the *P. multocida* serotype A- and D-stimulated groups, respectively (Fig. 4A, Tables S1 and S2). When the criterion was changed to —log_2_ (Fold-change)— > 0.1 and a *p-value* < 0.05, the differentially expressed miRNAs were screened out in both groups (Tables S3 and S4). As shown in Fig. 4B, contrary to the number of differentially expressed mRNAs, the number of upregulated miRNAs exceeded that of downregulated miRNAs.

**Figure 5:**
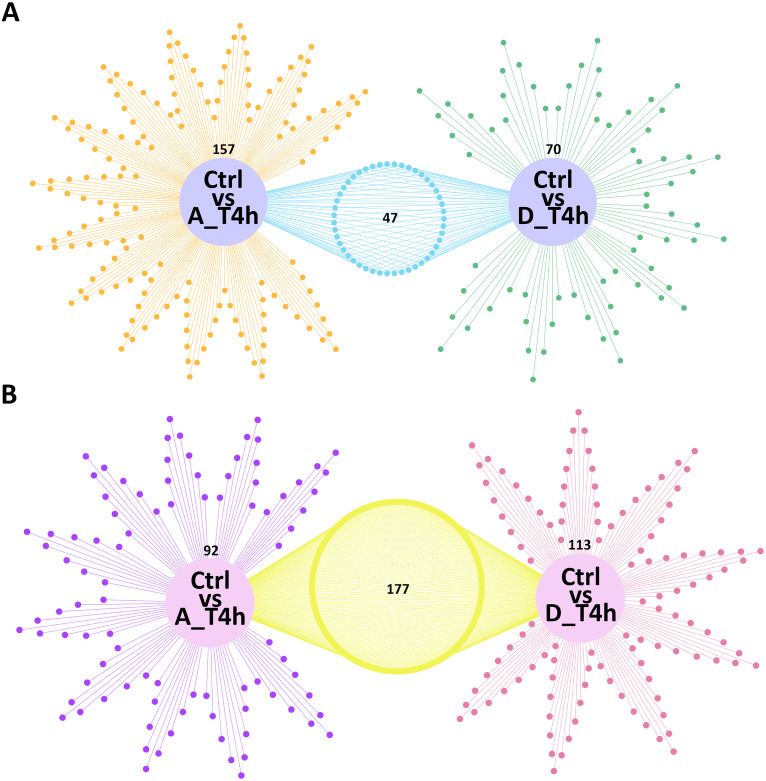
Venn network diagrams of the differentially expressed mRNAs (A) and miRNAs (B). (A) The orange (left) and green (right) dots represent the differentially expressed mRNAs unique to the Ctrl *vs* A_T4 h and Ctrl *vs* D_T4 h groups, respectively. The blue (middle) dots represent the differentially expressed mRNAs shared by the two groups. (B) The purple (left) and rose red (right) dots represent the differentially expressed miRNAs unique to the Ctrl *vs* A_T4 h and Ctrl *vs* D_T4 h groups, respectively. The yellow (middle) dots represent differentially expressed miRNAs shared by the two groups.

Venn network diagrams depicted the overlap among the differentially expressed mRNAs and miRNAs from the pairwise comparisons. In total, 157 and 70 specific intergroup differentially expressed mRNAs were detected in the *P. multocida* serotype A- and D-stimulated groups, respectively (Fig. 5A), compared with 92 and 113 differentially expressed miRNAs, respectively (Fig. 5B).

**Figure 6:**
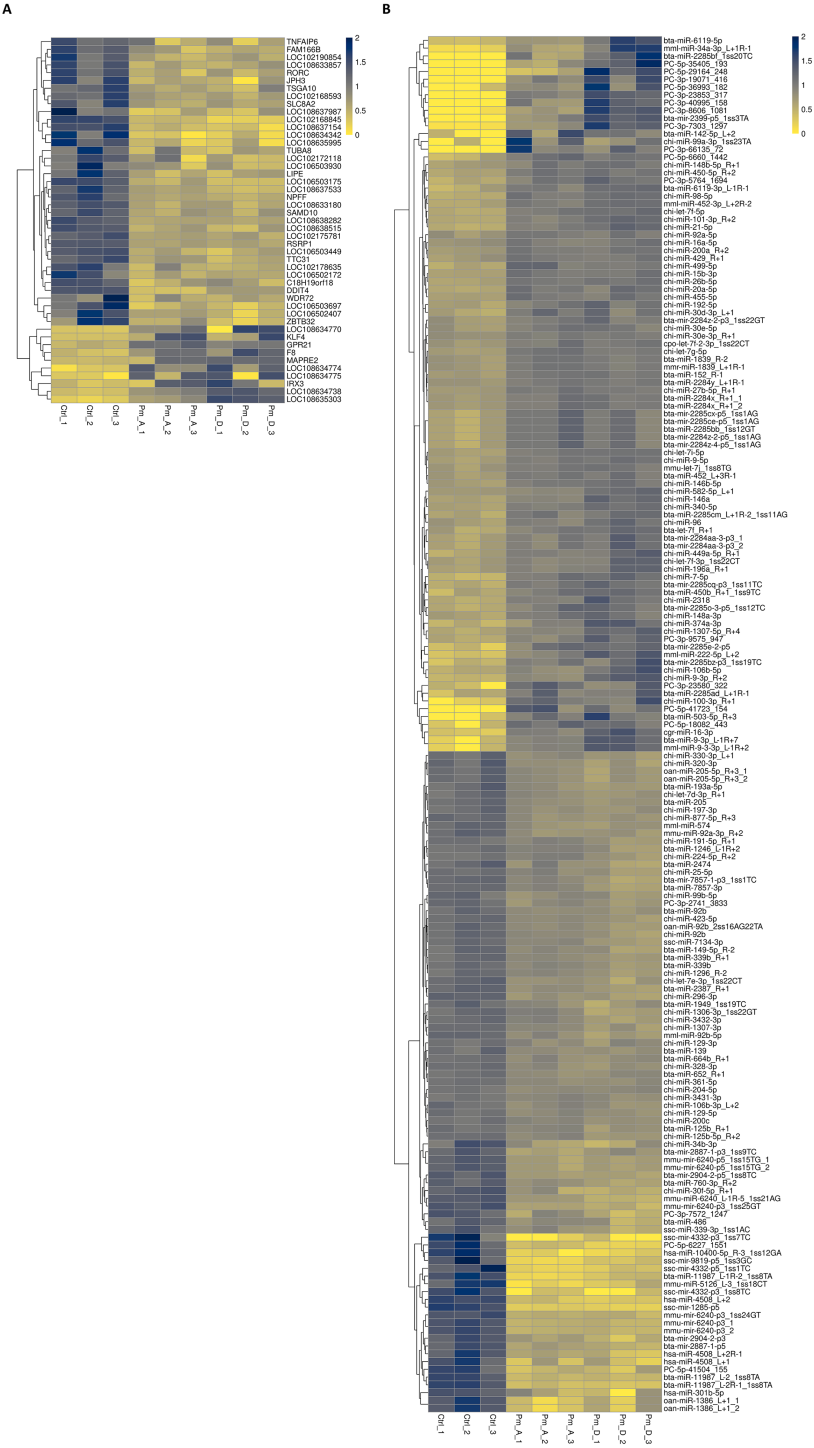
Heat map of common differentially expressed mRNAs (A) and miRNAs (B). (A) The FPKM of 47 differentially expressed mRNAs shared by the two stimulated groups were used to do the heat map. (B) The FPKM of 177 differentially expressed miRNAs shared by the two stimulated groups were used to do the heat map. The data were standardized with *z*-score.

Heat maps showed that most shared differentially expressed mRNAs were downregulated in expression (Fig. 6A), while only half of the miRNAs showed this trend (Fig. 6B).

### GO and KEGG pathway analyses

**Figure 7:**
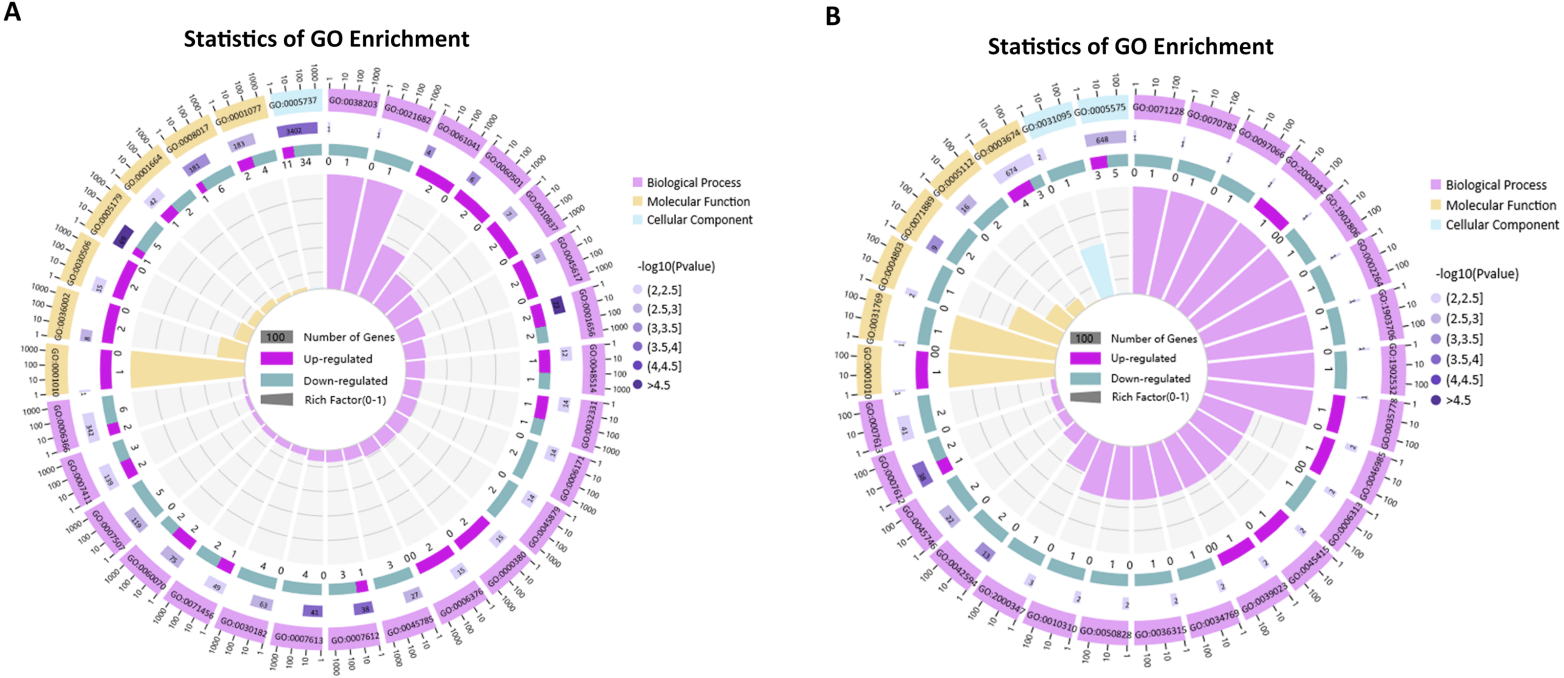
GO enrichment analysis of the differentially expressed mRNAs (top 30, *p-value*< 0.05). (A) GO enrichment analysis of the differentially expressed mRNAs in the *P. multocida* strain serotype A-stimulated group. (B) GO enrichment analysis of the differentially expressed mRNAs in the *P. multocida* strain serotype D-stimulated group.

As shown in Fig. 7, among the top 30 GO terms in each comparison group, the differentially expressed mRNAs were mainly enriched in biological processes. In the *P. multocida* strain serotype A-stimulated group, the GO terms with the most significant and the highest number of differentially expressed mRNAs were metanephros development (GO:0001656) and hormone activity (GO:0005179). The dysregulated mRNAs were mostly concentrated in GO terms that related to cell differentiation and proliferation (GO:0060501, GO:0010837, GO:0030182, GO:0045617, GO:0048514, GO:0032331). In the *P. multocida* strain serotype D-stimulated group, some differentially expressed mRNAs were annotated in immune responses (GO:0002264, GO:2000342, GO:0045415), oxidative stress (GO:0010310, GO:1903706, GO:0046985), and intracellular signal transduction (GO:1902532, GO:0097066, GO:0031769, GO:0036315, GO:0071889).

**Figure 8:**
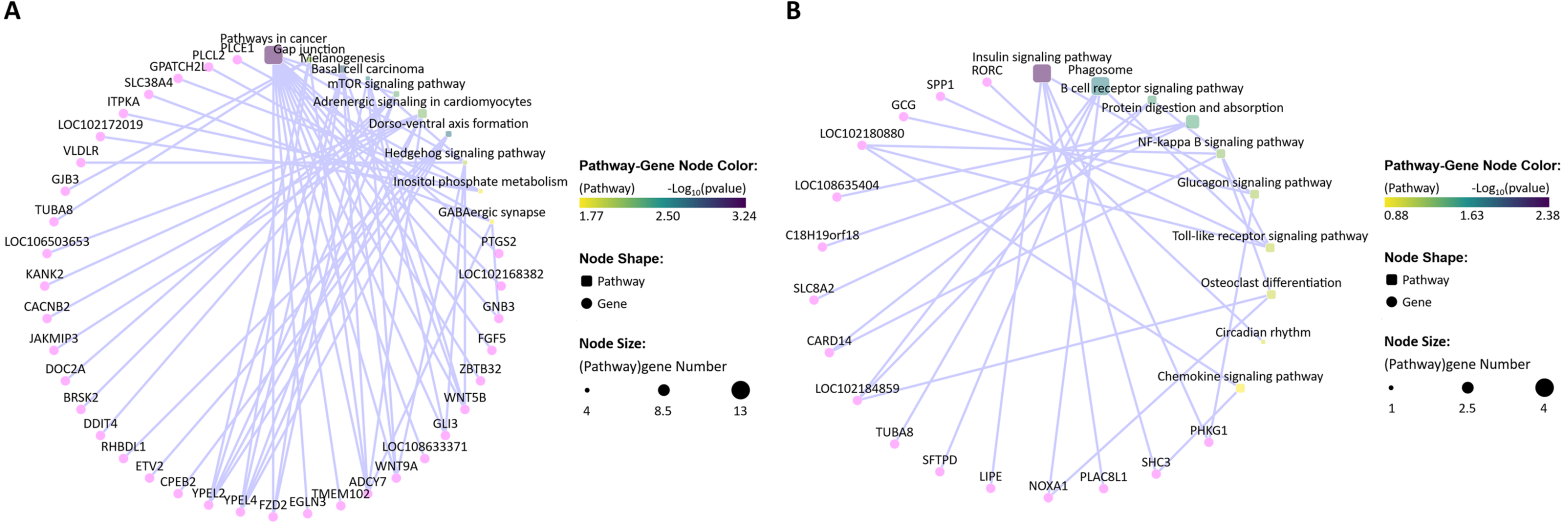
KEGG enrichment analysis of the differentially expressed mRNAs (top 10). (A) KEGG enrichment analysis of the differentially expressed mRNAs in the *P. multocida* strain serotype A-stimulated group. (B) KEGG enrichment analysis of the differentially expressed mRNAs in the *P. multocida* strain serotype D-stimulated group.

Through KEGG analysis, the most significantly enriched pathways for the dysregulated mRNAs in the *P. multocida* strain serotype A- and D-stimulated groups were pathways involved in cancer and insulin signaling (*p-value* < 0.05), respectively (Fig. 8). Compared with the *P. multocida* strain serotype D-stimulated group, the number of differentially expressed mRNAs enriched in the significant pathways was higher in the serotype A-stimulated group. However, the dysregulated mRNAs in the serotype D-stimulated group were significantly enriched in immune related pathways.

### qRT-PCR validation

**Figure 9:**
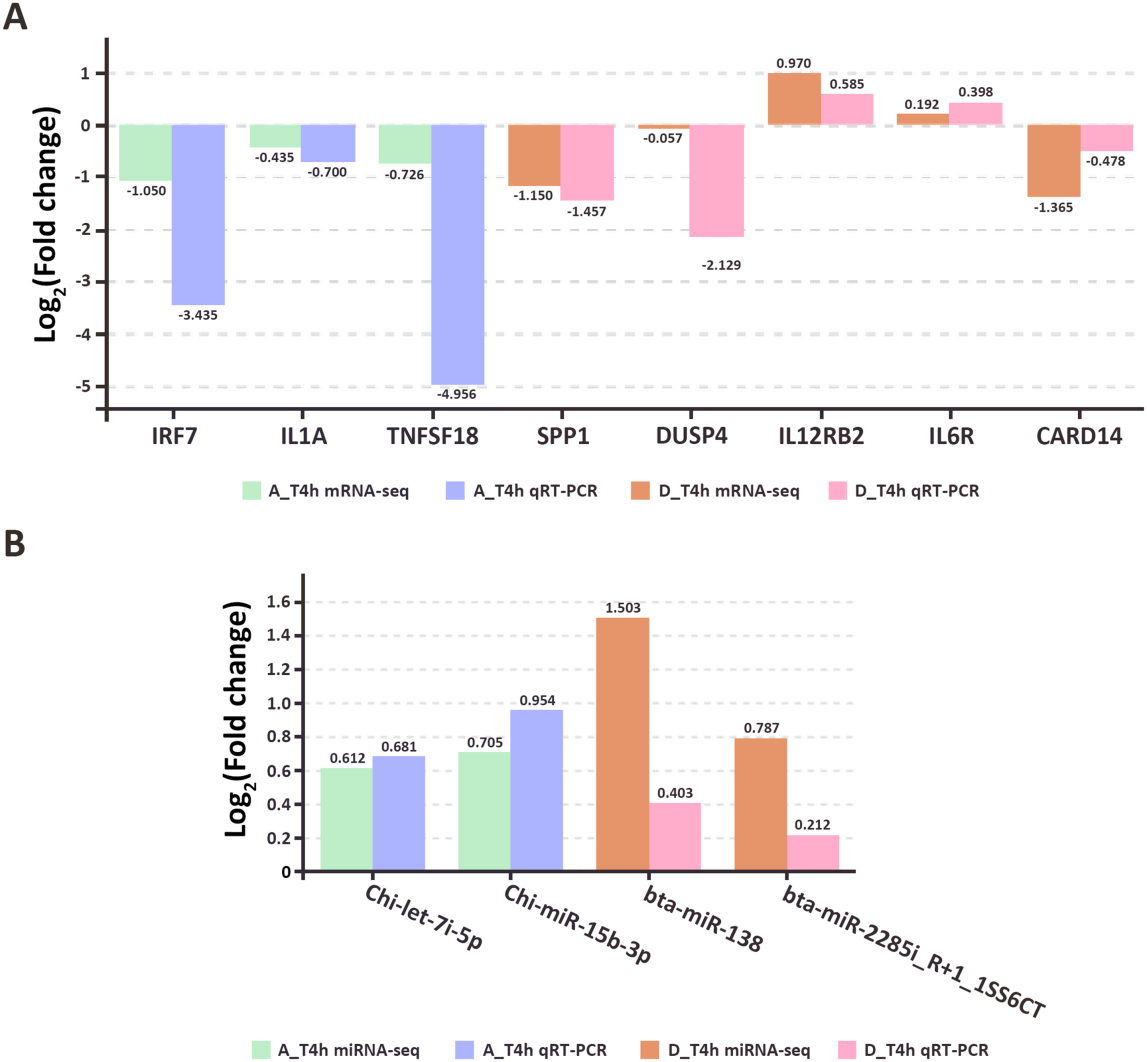
The qRT-PCR results of randomly selected genes (A) and miRNAs (B).

To assess the accuracy and reliability of the high-throughput sequencing results, eight genes (*IRF7, IL1A, TNFSF18, SPP1, DUSP4, IL12RB2, IL6R, CARD14*) and four miRNAs (*Chi-let-7i-5p, Chi-miR-15b-3p, bta-miR-138, bta-miR-2285i_R+1_1SS6CT*) were randomly selected for qRT-PCR validation. The results confirmed the correlation between the high-throughput sequencing data and the qRT-PCR results (Fig. 9, Table S5).

### Correlation analysis of mRNAs and miRNAs

**Figure 10:**
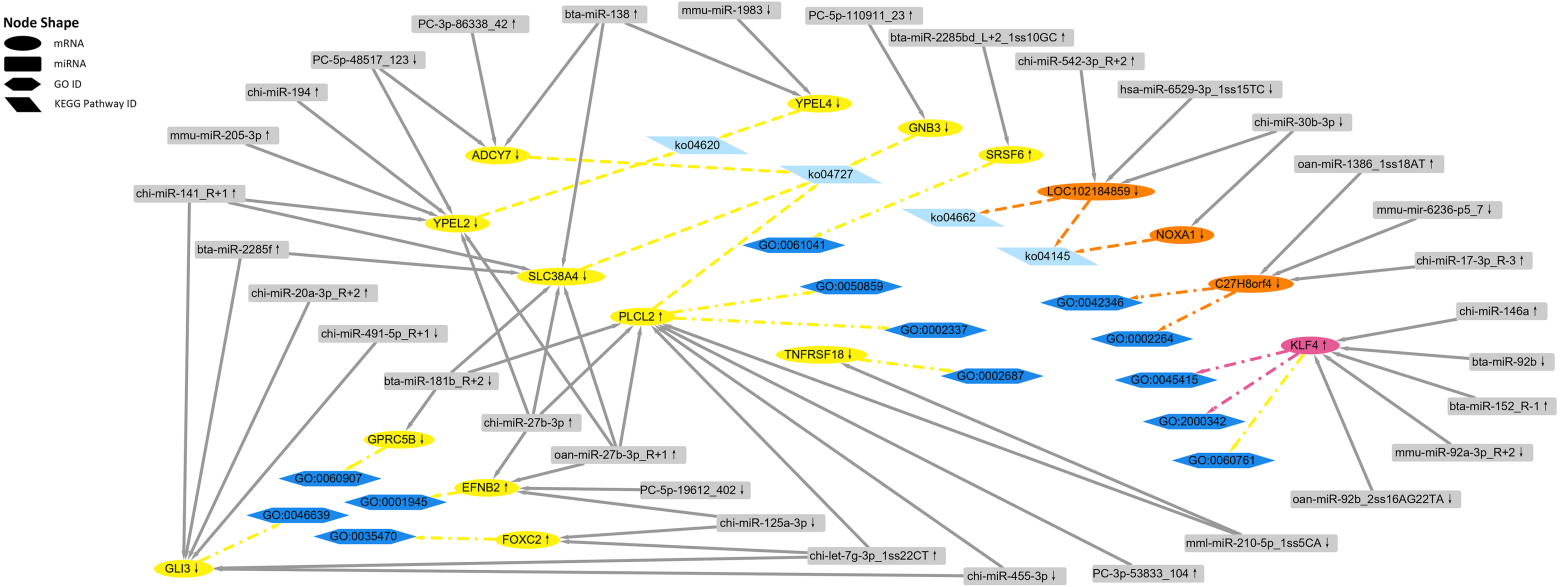
The regulatory networks of the target differentially expressed mRNAs and miRNAs. The yellow ellipses represent the differentially expressed mRNAs in the *P. multocida* strain serotype A-stimulated group. The orange ellipses represent the differentially expressed mRNAs in the *P. multocida* strain serotype D-stimulated group. The rose red ellipse represents the differentially expressed mRNA in both of the two stimulated groups. The gray rectangles represent the predicted differentially expressed miRNAs that target to the above mRNAs. The dark blue hexagons and light blue parallelograms represent enrichment to immune-related GO term (*p-value* < 0.05) and KEGG Pathways (*p-value* < 0.05), respectively. The yellow and orange lines represent the GO terms and KEGG Pathways enriched in *P. multocida* strain serotype A and D stimulated groups, respectively. The rose red lines represent the GO terms enriched in both of the two stimulated groups. The arrows after the mRNA and miRNA names indicate upregulated/downregulated expression.

According to the immune-related GO (*p-value* < 0.05) and KEGG (*p-value* < 0.05) enrichment results and the targeted relationship between miRNA and mRNA (Table S6–S13), 15 dysregulated mRNAs and 32 dysregulated miRNAs were selected to build the mRNA–miRNA network (Fig. 10). Through the network diagram, we discovered that one common gene (*KLF4*), which was differentially expressed in the two comparison groups, was annotated as negative regulation of the interleukin-8 biosynthetic process (GO:0045415), negative regulation of chemokine (C-X-C motif) ligand 2 production (GO:2000342), and negative regulation of the response to cytokine stimulus (GO:0060761). The first two GO terms were significant in both the *P. multocida* strain serotype A- and D-stimulated groups, while the other GO term was significant only in the serotype A-stimulated group. In addition, the dysregulated gene *C27H8orf4*, which was unique to the serotype D-stimulated group, was annotated as endothelial cell activation involved in the immune response (GO:0002264) and positive regulation of NF-kappa B imported into the nucleus (GO:0042346), while another unique gene *LOC102184859* was enriched in the B cell receptor signaling pathway (ko04662). Regarding the dysregulated genes specific to the serotype A-stimulated group, *PLCL2* predicted the largest number of miRNAs, which were enriched in the negative regulation of the B cell receptor signaling pathway (GO:0050859), B-1a B cell differentiation (GO:0002337), and GABAergic synapse (ko04727).

## Discussion

We first observed morphological changes in goat epithelial cells over different time periods and different doses when detecting the degree of damage caused to the cells by invading bacteria. When the stimulant was the *P. multocida* strain of serotype A at a MOI of 10 and the stimulation time was 2 h, the stimulated goat epithelial cells adhered well, but the intercellular spaces became larger. After 4 to 6 h of incubation, a large number of cells detached and cell death was observed, along with a significant increase in the number of *P. multocida*. After culturing for a further 2 h, the cell death rate increased and only a small number of cells remained adherent. At a MOI of 100 and a stimulation time of 2 to 4 h, the cells became rounded and detached, some cells died, and the number of *P. multocida* increased After 6 to 8 h of infection, almost no normal cells were observed, along with significant multiplication of *P. multocida*. At a MOI of 500, significant cell death was observed within 2 to 4 h of infection, and only a few cells remained adherent. After 8 h of infection, the number of *P. multocida* cells was significantly higher than that at a MOI of 100. When the stimulant was the *P. multocida* strain of serotype D, the cell changes were similar to those observed for the *P. multocida* serotype A strain. Therefore, we selected a MOI of 100 and a stimulation time of 4 h as the infection conditions for subsequent experiments.

MiRNAs are known to either inhibit translation of target mRNAs or facilitate their deadenylation and subsequent degradation (Fabian, Sonenberg & Filipowicz, 2010). In both stimulation groups, the number of downregulated mRNAs was significantly higher than upregulated mRNAs, while miRNAs showed the opposite trend (Fig. 4). Although several miRNAs are associated with the increased expression of target mRNAs (Stavast & Erkeland, 2019), mammalian miRNAs primarily act to reduce target mRNA levels (Guo et al., 2010). Our statistical results again supported the regulatory effect of miRNAs on target mRNAs.

In the *P. multocida* strain serotype A-stimulated group, the significant KEGG pathways related to immunity were GABAergic synapse (ko04727) and the Toll-like receptor signaling pathway (ko04620). A recent study reported that GABAergic neurotransmission plays an important role in central nervous system physiology and cellular immune regulation, and revealed a new “synapse—muscular insulin—intestinal innate immunity” signaling axis, which may help to maintain immunological homeostasis and promote host survival (Zheng et al., 2021). As shown in Fig. 10, *GNB3* (G protein subunit beta 3), *ADCY7* (adenylate cyclase 7), *SLC38A4* (solute carrier family 38 member 4), and *PLCL2* (phospholipase C like 2) were enriched in the GABAergic synapse pathway. With the exception of *PLCL2*, the other three genes were downregulated in expression in the *P. multocida* strain serotype A treatment group compared with the control group. GNB3 is an isoform of the heterotrimeric G protein *β* subunit and an important mediator of transmembrane signaling (Ye et al., 2014; Ford et al., 1998). A previous study reported that *GNB3* polymorphisms are associated with lung disease (Buroker et al., 2010). He and colleagues further demonstrated that *GNB3* polymorphisms may protect against high altitude pulmonary edema progression (He et al., 2017). Since *P. multocida* mainly causes pneumonia in goats and sheep, *GNB3* may also play a role in bacterial pneumonia. ADCY7 protein is one of a family of 10 enzymes that convert ATP to the ubiquitous second messenger cAMP, which regulates innate and adaptive immune functions (Luo et al., 2017). Cruz and colleagues confirmed that ADCY7 is important in corticotropin releasing factor modulation of presynaptic GABA release in the central amygdala (Cruz et al., 2011). In addition, inhibition of *ADCY7* expression upregulates the expression of immune-related genes (Duan et al., 2010; Jiang, Sternweis & Wang, 2013; Risøe et al., 2015). In the current study, the expression of *ADCY7* in goat bronchial epithelial cells was downregulated following stimulation with the *P. multocida* strain of serotype A. Downregulation of *ADCY7* may activate the GABAergic synapse pathway, potentially inducing the host immune response. Research has shown that *SLC38A4* depletion can repress hepatocellular carcinoma tumorigenesis *in vivo* (Li et al., 2021b). Furthermore, in a polymorphism study, *SLC38A4* rs2429467C >T consistently showed significant associations with lung cancer (Lee et al., 2016). Therefore, SLC38A4 may play a crucial role in pneumonia caused by *P. multocida* infection. *PLCL2* has been identified as a susceptibility gene (Arismendi et al., 2015), so inhibition of *PLCL2* expression may suppress the infection of *P. multocida*.

In addition, 11 differentially expressed miRNAs were identified in the current study that may regulate *GNB3, ADCY7, SLC38A4*, and *PLCL2* (Fig. 10), seven of which were upregulated. The expression levels of targeted mRNAs and miRNAs usually show the opposite trend. Ten pairs of mRNA–miRNAs conformed this regulatory relationship, and *bta-miR-138* was the only miRNA that simultaneously regulated two genes (*ADCY7* and *SLC38A4*). By qRT-PCR, Singh and colleagues verified that the expression of *bta-miR-138* in peripheral blood mononuclear cells stimulated by Toll-like receptors (TLRs) was increased (Singh et al., 2016). Our sequencing results confirmed that the expression of *bta-miR-138* was significantly increased after *P. multocida* strain serotype A stimulation. According to the mRNA–miRNA network diagram, *YPEL4* (yippee-like 4) was also a target gene of *bta-miR-138*, and this gene was enriched in the TLR signaling pathway. Therefore, *bta-miR-138* may play a role in the immune response induced by *P. multocida*.

*YPEL2* (yippee-like 2) was also enriched in the TLR signaling pathway. TLRs are protective immune sentries that are known for their role in innate immunity, detecting and defending against microbial pathogens (Lim & Staudt, 2013; Brennan & Gilmore, 2018). *YPEL2* and *YPEL4* annotated to this pathway are both members of the *YPEL* (yippee-like) gene family, which are highly conserved across a diverse range of eukaryotic organisms (Mattebo et al., 2021). However, there are few studies on *YPEL2*. *YPEL4* is part of a complex network of pathways involved in the development of many pulmonary diseases (Truong et al., 2018). The *P. multocida* serotype A strain may lead to downregulation of *YPEL4* through TLRs, implying its involvement in pneumonia.

In the *P. multocida* strain serotype D-stimulated group, the significant KEGG pathways related to immunity were the phagosome (ko04145) and B cell receptor signaling pathway (ko04662). Notably, *LOC102184859* was enriched in both pathways, and only the expression of *chi-mir-542-3P_R* +2 was upregulated, which was consistent with the regulatory relationship between mRNAs and miRNAs (Fig. 10). However, the function of *LOC102184859* and *chi-mir-542-3P* remain largely unknown, and further studies are needed to investigate their immunological roles.

Interestingly, the number of significantly regulated KEGG pathways for the *P. multocida* strain serotype A-stimulated group was higher than that for the serotype D-stimulated group, and most were related to cell morphology, proliferation, differentiation, and signal transduction. Among these pathways, only GABAergic synapse and the TLR signaling pathway were associated with immunity. The former is a newly revealed pathway that may be associated with immunity, while the latter is a well-known immune-related KEGG pathway. TLRs are the first line of defense against pathogen invasion. However, the significant KEGG pathways for the *P. multocida* strain serotype D-stimulated group were classic immune-related pathways (phagosome and B cell receptor signaling pathway). Phagosomes are an indispensable component of the immune response to eliminate invading bacteria and viruses (Omotade & Roy, 2019). Antigen recognition by B cell receptors is the first step for T cells in helping naive B cells differentiate into plasma cells, germinal center B cells, and memory B cells (Tanaka & Baba, 2020). These results are consistent with the observed cellular changes. We hypothesized that when the *P. multocida* serotype A strain stimulated goat bronchial epithelial cells for 4 h, TLRs induced a series of intracellular signal transduction events that caused morphological changes in cells. The GABAergic synapse received the stimulating signal, but the immune response had not yet been induced. Whereas in the *P. multocida* strain serotype D-stimulated group, the immune response had already been induced. Thus, the significant KEGG pathways related to cell morphology were decreased, and the observed changes in cell morphology were more obvious. This also indirectly suggests that serotype D of *P. multocida* may be more virulent than serotype A.

## Conclusions

Our research demonstrated transcriptome changes, as determined by high-throughput sequencing, when serotype A and D strains of *P. multocida* were used to stimulate goat bronchial epithelial cells. A total of 204 and 117 dysregulated mRNAs were identified in the *P. multocida* strain serotype A- and D-stimulated groups, respectively, compared with 269 and 290 dysregulated miRNAs. The significant KEGG pathways related to immunity were GABAergic synapse, Toll-like receptor signaling pathway, and B cell receptor signaling pathway. Serotype D *P. multocida* may thereby induce a cellular immune response earlier than serotype A in stimulated goat bronchial epithelial cells.

##  Supplemental Information

10.7717/peerj.13047/supp-1 Table S1 Total differentially expressed genes in the *P. multocida* strain serotype A- stimulated group

10.7717/peerj.13047/supp-2 Table S2 Total differentially expressed genes in the *P. multocida* strain serotype D- stimulated group

10.7717/peerj.13047/supp-3 Table S3 Total differentially expressed miRNAs in the *P. multocida* strain serotype A- stimulated group

10.7717/peerj.13047/supp-4 Table S4 Total differentially expressed miRNAs in the *P. multocida* strain serotype D- stimulated group

10.7717/peerj.13047/supp-5 Table S5 Analysis of qRT-PCR data

10.7717/peerj.13047/supp-6 Table S6 Prediction of miRNA target genes in the *P. multocida* strain serotype A- stimulated group

10.7717/peerj.13047/supp-7 Table S7 Prediction of miRNA target genes in the *P. multocida* strain serotype D- stimulated group

10.7717/peerj.13047/supp-8 Table S8 GO enrichment analysis of specific differentially expressed genes in the *P. multocida* strain serotype A- stimulated group

10.7717/peerj.13047/supp-9 Table S9 GO enrichment analysis of specific differentially expressed genes in the *P. multocida* strain serotype D -stimulated group

10.7717/peerj.13047/supp-10 Table S10 GO enrichment analysis of common differentially expressed genes in the *P. multocida* strain serotypes A- and D- stimulated groups

10.7717/peerj.13047/supp-11 Table S11 KEGG enrichment analysis of specific differentially expressed genes in the* P. multocida* strain serotype A-stimulated group

10.7717/peerj.13047/supp-12 Table S12 KEGG enrichment analysis of specific differentially expressed genes in the *P. multocida* strain serotype D- stimulated group

10.7717/peerj.13047/supp-13 Table S13 KEGG enrichment analysis of common differentially expressed genes in the *P. multocida* strain serotype A- and D-stimulated groups

10.7717/peerj.13047/supp-14 Figure S1 Growth curves (A) and standard linear equations (B) of *P. multocida* serotype A (A) The horizontal axis represents the incubation time of *P. multocida* strain serotype A in TSB. The vertical axis represents the absorption value of bacterial suspension at OD_600 nm_. (B) In the standard linear equation, *X* represents the absorption value at OD_600 nm_, and *Y* represents the CFU/mL.

10.7717/peerj.13047/supp-15 Figure S2 Growth curves (A) and standard linear equations (B) of *P. multocida* serotype D (A) The horizontal axis represents the incubation time of *P. multocida* strain serotype D in TSB. The vertical axis represents the absorption value of bacterial suspension at OD_600 nm_. (B) In the standard linear equation, *X* represents the absorption value at OD_600 nm_, and *Y* represents the CFU/mL.

## Additional Information and Declarations

### Data Availability

The following information was supplied regarding data availability: The raw reads are available at NCBI: PRJNA655134 and PRJNA655261.

## References

[ref-1] Aprianto R, Slager J, Holsappel S, Veening JW (2016). Time-resolved dual RNA-Seq reveals extensive rewiring of lung epithelial and pneumococcal transcriptomes during early infection. Genome Biology.

[ref-2] Arismendi M, Giraud M, Ruzehaji N, Dieudé P, Koumakis E, Ruiz B, Airo P, Cusi D, Matucci-Cerinic M, Salvi E, Cuomo G, Hachulla E, Diot E, Caramaschi P, Riccieri V, Avouac J, Kayser C, Allanore Y (2015). Identification of NF- *κ*B and PLCL2 as new susceptibility genes and highlights on a potential role of IRF8 through interferon signature modulation in systemic sclerosis. Arthritis Research & Therapy.

[ref-3] Boyce JD, Adler B (2000). The capsule is a virulence determinant in the pathogenesis of *Pasteurella multocida* M1404 (B:2). Infection and Immunity.

[ref-4] Brennan JJ, Gilmore TD (2018). Evolutionary origins of toll-like receptor signaling. Molecular Biology and Evolution.

[ref-5] Buroker NE, Ning XH, Zhou ZN, Li K, Cen WJ, Wu XF, Ge M, Fan LP, Zhu WZ, Portman MA, Chen SH (2010). Genetic associations with mountain sickness in Han and Tibetan residents at the Qinghai-Tibetan Plateau. Clinica Chimica Acta.

[ref-6] Cao R, Zhang Z, Nie X, Li B, Huang H, Yang X, Zhu S, Du L, Wang F (2018). Isolation, identification and phylogenetic analysis of *Pasteurella multocida*. Chinese Journal of Veterinary Medicine.

[ref-7] Cer RZ, Herrera-Galeano JE, Anderson JJ, Bishop-Lilly KA, Mokashi VP (2014). miRNA Temporal Analyzer (mirnaTA): a bioinformatics tool for identifying differentially expressed microRNAs in temporal studies using normal quantile transformation. Gigascience.

[ref-8] Chen Q, Zhang Z, Chen S, Chen J, Cheng Y, Liu A, Li B, Chen Z, Zheng Y, Ga M, Du L, Wang F (2021). Genome-wide differential expression profiling of pulmonary circRNAs associated with immune reaction to *Pasteurella multocida* in goats. Frontiers in Veterinary Science.

[ref-9] Cloonan N, Forrest AR, Kolle G, Gardiner BB, Faulkner GJ, Brown MK, Taylor DF, Steptoe AL, Wani S, Bethel G, Robertson AJ, Perkins AC, Bruce SJ, Lee CC, Ranade SS, Peckham HE, Manning JM, McKernan KJ, Grimmond SM (2008). Stem cell transcriptome profiling via massive-scale mRNA sequencing. Nature Methods.

[ref-10] Costa V, Angelini C, De Feis I, Ciccodicola A (2010). Uncovering the complexity of transcriptomes with RNA-Seq. Journal of Biomedicine and Biotechnology.

[ref-11] Cruz MT, Bajo M, Maragnoli ME, Tabakoff B, Siggins GR, Roberto M (2011). Type 7 Adenylyl Cyclase is involved in the ethanol and CRF sensitivity of GABAergic synapses in mouse central amygdala. Frontiers in Neuroscience.

[ref-12] Duan B, Davis R, Sadat EL, Collins J, Sternweis PC, Yuan D, Jiang LI (2010). Distinct roles of adenylyl cyclase VII in regulating the immune responses in mice. Journal of Immunology.

[ref-13] Fabian MR, Sonenberg N, Filipowicz W (2010). Regulation of mRNA translation and stability by microRNAs. Annual Review of Biochemistry.

[ref-14] Ford CE, Skiba NP, Bae H, Daaka Y, Reuveny E, Shekter LR, Rosal R, Weng G, Yang CS, Iyengar R, Miller RJ, Jan LY, Lefkowitz RJ, Hamm HE (1998). Molecular basis for interactions of G protein betagamma subunits with effectors. Science.

[ref-15] Guo H, Ingolia NT, Weissman JS, Bartel DP (2010). Mammalian microRNAs predominantly act to decrease target mRNA levels. Nature.

[ref-16] Harper M, Cox AD, Adler B, Boyce JD (2011). *Pasteurella multocida* lipopolysaccharide: the long and the short of it. Veterinary Microbiology.

[ref-17] Harper M, Cox A, Michael FSt, Parnas H, Wilkie I, Blackall PJ, Adler B, Boyce JD (2007). Decoration of *Pasteurella multocida* lipopolysaccharide with phosphocholine is important for virulence. Journal of Bacteriology.

[ref-18] He Y, Liu L, Xu P, He N, Yuan D, Kang L, Jin T (2017). Association between single nucleotide polymorphisms in ADRB2, GNB3 and GSTP1 genes and high-altitude pulmonary edema (HAPE) in the Chinese Han population. Oncotarget.

[ref-19] Heddleston KL, Gallagher JE, Rebers PA (1972). Fowl cholera: gel diffusion precipitin test for serotyping Pasteruella multocida from avian species. Avian Diseases.

[ref-20] Jiang LI, Sternweis PC, Wang JE (2013). Zymosan activates protein kinase A via adenylyl cyclase VII to modulate innate immune responses during inflammation. Molecular Immunology.

[ref-21] Kubatzky KF (2012). *Pasteurella multocida* and immune cells. Current Topics in Microbiology and Immunology.

[ref-22] Lee SY, Kang HG, Choi JE, Jung DK, Lee WK, Lee HC, Lee SY, Yoo SS, Lee J, Seok Y, Lee EB, Cha SI, Cho S, Kim CH, Lee MH, Park JY (2016). Polymorphisms in cancer-related pathway genes and lung cancer. European Respiratory Journal.

[ref-23] Li B, Chen S, Wang C, Chen Q, Man C, An Q, Zhang Z, Liu Z, Du L, Wang F (2021a). Integrated mRNA-seq and miRNA-seq analysis of goat fibroblasts response to Brucella Melitensis strain M5-90. PeerJ.

[ref-24] Li J, Li MH, Wang TT, Liu XN, Zhu XT, Dai YZ, Zhai KC, Liu YD, Lin JL, Ge RL, Sun SH, Wang F, Yuan JH (2021b). SLC38A4 functions as a tumour suppressor in hepatocellular carcinoma through modulating Wnt/ *β*-catenin/MYC/HMGCS2 axis. British Journal of Cancer.

[ref-25] Li X, Shahid MQ, Wu J, Wang L, Liu X, Lu Y (2016). Comparative small RNA analysis of pollen development in autotetraploid and diploid rice. International Journal of Molecular Sciences.

[ref-26] Lim KH, Staudt LM (2013). Toll-like receptor signaling. Cold Spring Harbor Perspectives in Biology.

[ref-27] Luo Y, De Lange KM, Jostins L, Moutsianas L, Randall J, Kennedy NA, Lamb CA, McCarthy S, Ahmad T, Edwards C, Serra EG, Hart A, Hawkey C, Mansfield JC, Mowat C, Newman WG, Nichols S, Pollard M, Satsangi J, Simmons A, Tremelling M, Uhlig H, Wilson DC, Lee JC, Prescott NJ, Lees CW, Mathew CG, Parkes M, Barrett JC, Anderson CA (2017). Exploring the genetic architecture of inflammatory bowel disease by whole-genome sequencing identifies association at ADCY7. Nature Genetics.

[ref-28] Marques R, Boneca IG (2011). Expression and functional importance of innate immune receptors by intestinal epithelial cells. Cellular and Molecular Life Sciences.

[ref-29] Mattebo A, Sen T, Jassinskaja M, Pimková K, Prieto González-Albo I, Alattar AG, Ramakrishnan R, Lang S, Järås M, Hansson J, Soneji S, Singbrant S, Van den Akker E, Flygare J (2021). Yippee like 4 (Ypel4) is essential for normal mouse red blood cell membrane integrity. Scientific Reports.

[ref-30] Mogilner L, Katz C (2019). Pasteurella multocida. Pediatric Review.

[ref-31] Morin R, Bainbridge M, Fejes A, Hirst M, Krzywinski M, Pugh T, McDonald H, Varhol R, Jones S, Marra M (2008). Profiling the HeLa S3 transcriptome using randomly primed cDNA and massively parallel short-read sequencing. Biotechniques.

[ref-32] Nagalakshmi U, Wang Z, Waern K, Shou C, Raha D, Gerstein M, Snyder M (2008). The transcriptional landscape of the yeast genome defined by RNA sequencing. Science.

[ref-33] Omotade TO, Roy CR (2019). Manipulation of host cell organelles by intracellular pathogens. Microbiology Spectrum.

[ref-34] Pang F, Zhang M, Yang X, Li G, Zhu S, Nie X, Cao R, Yang X, Zhang Z, Huang H, Li B, Wang C, Du L, Wang F (2019). Genome-wide analysis of circular RNAs in goat skin fibroblast cells in response to Orf virus infection. PeerJ.

[ref-35] Peng Z, Wang X, Zhou R, Chen H, Wilson BA, Wu B (2019). *Pasteurella multocida*: genotypes and genomics. Microbiology and Molecular Biology Reviews.

[ref-36] Risøe PK, Rutkovskiy A, Ågren J, Kolseth IB, Kjeldsen SF, Valen G, Vaage J, Dahle MK (2015). Higher TNF *α* responses in young males compared to females are associated with attenuation of monocyte adenylyl cyclase expression. Human Immunology.

[ref-37] Sellyei B, Bányai K, Magyar T (2010). Characterization of the ptfA gene of avian *Pasteurella multocida* strains by allele-specific polymerase chain reaction. Journal of Veterinary Diagnostic Investigation.

[ref-38] Singh J, Mukhopadhyay CS, Kaur S, Malhotra P, Sethi RS, Choudhary RK (2016). Identification of the MicroRNA repertoire in TLR-ligand challenged bubaline PBMCs as a model of bacterial and viral infection. PLOS ONE.

[ref-39] Stavast CJ, Erkeland SJ (2019). The non-canonical aspects of MicroRNAs: many roads to gene regulation. Cells.

[ref-40] Sudaryatma PE, Nakamura K, Mekata H, Sekiguchi S, Kubo M, Kobayashi I, Subangkit M, Goto Y, Okabayashi T (2018). Bovine respiratory syncytial virus infection enhances *Pasteurella multocida* adherence on respiratory epithelial cells. Veterinary Microbiology.

[ref-41] Tanaka S, Baba Y (2020). B cell receptor signaling. Advances in Experimental Medicine and Biology.

[ref-42] Tang X, Zhao Z, Hu J, Wu B, Cai X, He Q, Chen H (2009). Isolation, antimicrobial resistance, and virulence genes of *Pasteurella multocida* strains from swine in China. Journal of Clinical Microbiology.

[ref-43] Truong L, Zheng YM, Song T, Tang Y, Wang YX (2018). Potential important roles and signaling mechanisms of YPEL4 in pulmonary diseases. Clinical and Translational Medicine.

[ref-44] Wang Z, Gerstein M, Snyder M (2009). RNA-Seq: a revolutionary tool for transcriptomics. Nature Reviews Genetics.

[ref-45] Wilkie IW, Harper M, Boyce JD, Adler B (2012). *Pasteurella multocida*: diseases and pathogenesis. Current Topics in Microbiology and Immunology.

[ref-46] Wilson BA, Ho M (2013). *Pasteurella multocida*: from zoonosis to cellular microbiology. Clinical Microbiology Reviews.

[ref-47] Wu C, Qin X, Li P, Pan T, Ren W, Li N, Peng Y (2017). Transcriptomic analysis on responses of murine lungs to *Pasteurella multocida* infection. Frontiers in Cellular and Infection Microbiology.

[ref-48] Ye Y, Sun Z, Guo A, Song LS, Grobe JL, Chen S (2014). Ablation of the GNB3 gene in mice does not affect body weight, metabolism or blood pressure, but causes bradycardia. Cell Signaling.

[ref-49] Zamri-Saad M, Effendy WM, Maswati MA, Salim N, Sheikh-Omar AR (1996). The goat as a model for studies of pneumonic pasteurellosis caused by *Pasteurella multocida*. British Veterinary Journal.

[ref-50] Zheng Z, Zhang X, Liu J, He P, Zhang S, Zhang Y, Gao J, Yang S, Kang N, Afridi MI, Gao S, Chen C, Tu H (2021). GABAergic synapses suppress intestinal innate immunity via insulin signaling in Caenorhabditis elegans. Proceedings of the National Academy of Sciences of the United States of America.

