# Supplementary figures and images for: The mRNA and miRNA profiles of goat bronchial epithelial cells stimulated by Pasteurella multocida strains of serotype A and D
Source: PeerJ. 2022 Mar 18;10:e13047. doi: 10.7717/peerj.13047 (PMC8935994; doi:10.7717/peerj.13047)

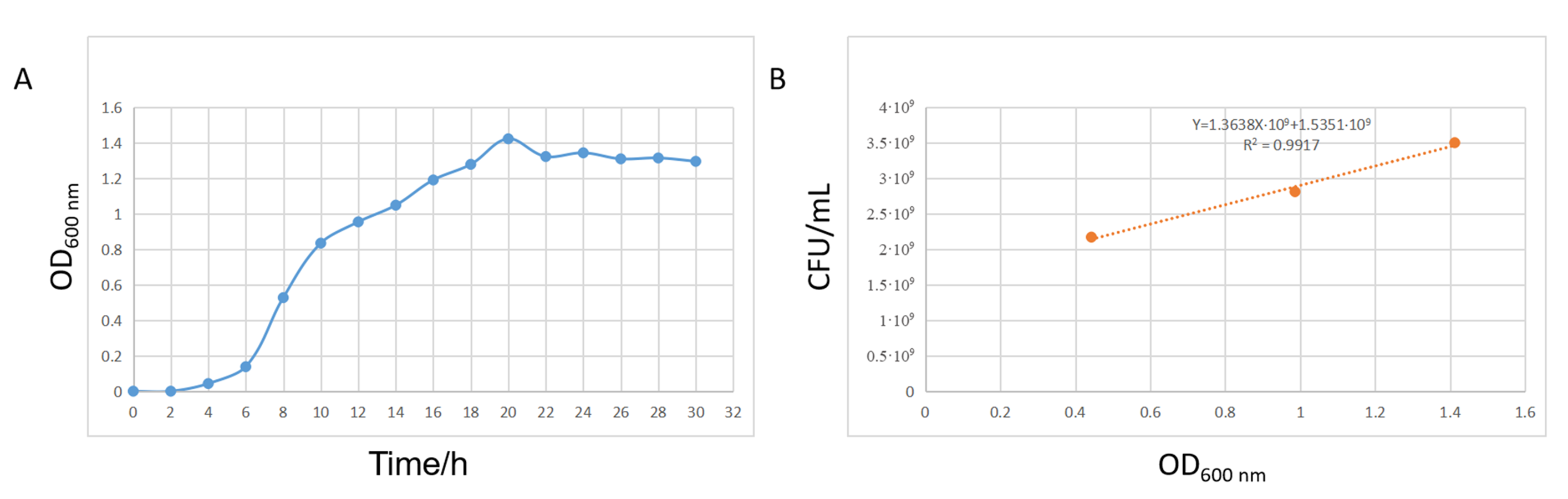

Supplement: Figure S1 — (A) The horizontal axis represents the incubation time of P. multocida strain serotype A in TSB. The vertical axis represents the absorption value of bacterial suspension at OD600 nm. (B) In the standard linear equation, X represents the absorption value at OD600 nm, and Y represents the CFU/mL. [file peerj-10-13047-s014.png]

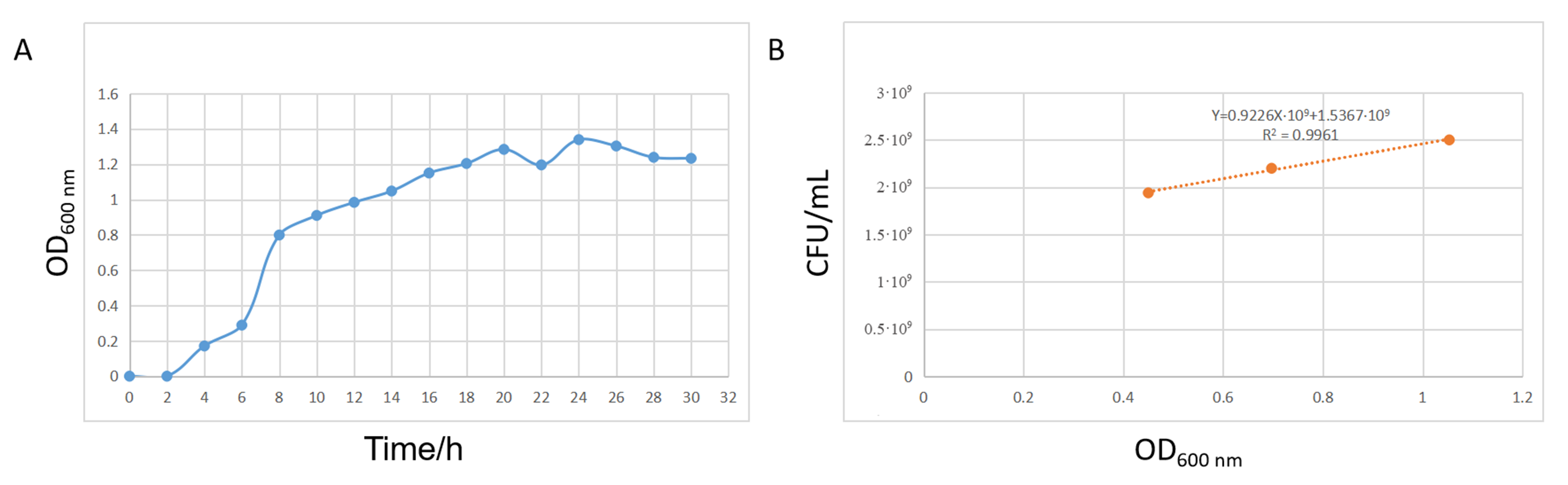

Supplement: Figure S2 — (A) The horizontal axis represents the incubation time of P. multocida strain serotype D in TSB. The vertical axis represents the absorption value of bacterial suspension at OD600 nm. (B) In the standard linear equation, X represents the absorption value at OD600 nm, and Y represents the CFU/mL. [file peerj-10-13047-s015.png]
